# Tracheostomy in patients with acute respiratory distress syndrome is not related to quality of life, symptoms of psychiatric disorders or return-to-work: the prospective DACAPO cohort study

**DOI:** 10.1186/s13613-020-00671-x

**Published:** 2020-05-06

**Authors:** Sebastian Blecha, Magdalena Brandl, Florian Zeman, Frank Dodoo-Schittko, Susanne Brandstetter, Christian Karagiannidis, Thomas Bein, Christian Apfelbacher, for the DACAPO Study Group, Johannes Bickenbach, Thorben Beeker, Tobias Schürholz, Jessica Pezechk, Jens Schloer, Ulrich Jaschinski, Ilse Kummer, Oliver Kuckein, Steffen Weber-Carstens, Anton Goldmann, Stefan Angermair, Krista Stoycheva, Jörg Brederlau, Nadja Rieckehr, Gabriele Schreiber, Henriette Haennicke, Friedhelm Bach, Immo Gummelt, Silke Haas, Catharina Middeke, Ina Vedder, Marion Klaproth, Michael Adamzik, Jan Karlik, Stefan Martini, Luisa Robitzky, Christian Putensen, Thomas Muders, Ute Lohmer, Rolf Dembinski, Petra Schäffner, Petra Wulff-Werner, Elke Landsiedel-Mechenbier, Daniela Nickoleit-Bitzenberger, Ann-Kathrin Silber, Maximilian Ragaller, Marcello Gama de Abreu, Alin Ulbricht, Linda Reisbach, Kai Zacharowski, Patrick Meybohm, Alexander Hötzel, Johannes Kalbhenn, Christoph Metz, Stefan Haschka, Stefan Rauch, Michael Quintel, Lars-Olav Harnisch, Sophie Baumann, Andrea Kernchen, Sigrun Friesecke, Sebastian Maletzki, Stefan Kluge, Olaf Boenisch, Daniel Frings, Birgit Füllekrug, Nils Jahn, Knut Kampe, Grit Ringeis, Brigitte Singer, Robin Wüstenberg, Jörg Ahrens, Heiner Ruschulte, Andre Gerdes, Matthias Groß, Olaf Wiesner, Aleksandra Bayat-Graw, Thorsten Brenner, Felix Schmitt, Anna Lipinski, Dietrich Henzler, Klaas Eickmeyer, Juliane Krebs, Iris Rodenberg, Heinrich Groesdonk, Kathrin Meiers, Karen Salm, Thomas Volk, Stefan Fischer, Basam Redwan, Martin Schmölz, Kathrin Schumann-Stoiber, Simone Eberl, Gunther Lenz, Thomas von Wernitz-Keibel, Monika Zackel, Frank Bloos, Petra Bloos, Anke Braune, Anja Haucke, Almut Noack, Steffi Kolanos, Heike Kuhnsch, Karina Knuhr-Kohlberg, Markus Gehling, Mathias Haller, Anne Sturm, Jannik Rossenbach, Dirk Schädler, Stefanie D’Aria, Christian Karagiannidis, Stephan Straßmann, Wolfram Windisch, Thorsten Annecke, Holger Herff, Michael Schütz, Sven Bercker, Hannah Reising, Mandy Dathe, Christian Schlegel, Katrin Lichy, Wolfgang Zink, Jana Kötteritzsch, Marc Bodenstein, Susanne Mauff, Peter Straub, Christof Strang, Florian Prätsch, Thomas Hachenberg, Thomas Kirschning, Thomas Friedrich, Dennis Mangold, Christian Arndt, Tilo Koch, Hendrik Haake, Katrin Offermanns, Patrick Friederich, Florian Bingold, Michael Irlbeck, Bernhard Zwissler, Ines Kaufmann, Ralph Bogdanski, Barbara Kapfer, Markus Heim, Günther Edenharter, Björn Ellger, Daniela Bause, Götz Gerresheim, Dorothea Muschner, Michael Christ, Arnim Geise, Martin Beiderlinden, Thorsten Heuter, Alexander Wipfel, Werner Kargl, Marion Harth, Christian Englmeier, Thomas Bein, Sebastian Blecha, Kathrin Thomann-Hackner, Marius Zeder, Markus Stephan, Martin Glaser, Helene Häberle, Hendrik Bracht, Christian Heer, Theresa Mast, Markus Kredel, Ralf Müllenbach, Phillip Sebök, Kathrin Thomann-Hackner, Julika Loss, Bernhard Graf, Michael Leitzmann, Michael Pfeifer, Simon Bein, Vreni Brunnthaler, Carina Forster, Stefanie Hertling, Sophie Höhne, Carolin Schimmele, Elisa Valletta

**Affiliations:** 1 Department of Anaesthesiology, University Medical Centre Regensburg, Franz-Josef-Strauss-Allee 11, 93053 Regensburg, Germany; 2 Medical Sociology, Institute of Epidemiology and Preventive Medicine, University of Regensburg, Regensburg, Germany; 3 Centre of Clinical Studies, University Medical Centre Regensburg, Regensburg, Germany; 4 University Children‘s Hospital Regensburg (KUNO), University of Regensburg, Regensburg, Germany; 5 Department of Pneumology and Critical Care Medicine, Cologne-Merheim Hospital, ARDS and ECMO Centre, Kliniken der Stadt Köln gGmbH, Witten/Herdecke University Hospital, Cologne, Germany; 6 Institute of Social Medicine and Health Economics, Otto von Guericke University Magdeburg, Magdeburg, Germany

**Keywords:** Tracheostomy, ARDS, Intensive care, Health-related quality of life, Return-to-work

## Abstract

**Background:**

Acute respiratory distress syndrome (ARDS) is a life-threatening condition that often requires prolonged mechanical ventilation. Tracheostomy is a common procedure with some risks, on the other hand with potential advantages over orotracheal intubation in critically ill patients. This study investigated the association of tracheostomy with health-related quality of life (HRQoL), symptoms of psychiatric disorders and return-to-work of ARDS survivors.

**Methods:**

Data were collected in the context of the prospective observational German-wide DACAPO study. Clinical and demographic patient data and treatment characteristics were obtained from the participating intensive care units (ICU). HRQoL and return-to-work were assessed using patient-reported questionnaires 3, 6 and 12 months after ICU discharge. HRQoL was measured with the Physical and Mental Component Scale of the Short-Form 12 Questionnaire (PCS-12, MCS-12). The prevalence of psychiatric symptoms (depression and post-traumatic stress disorder [PTSD]) was assessed using the Patient Health Questionnaire-9 and the Post-Traumatic Stress Syndrome-14. Physician-diagnosed anxiety and obsessive–compulsive disorder were recorded by patient self-report in the follow-up questionnaires. The associations of tracheostomy with HRQoL, psychiatric symptoms and return-to-work after 12 months were investigated by means of multivariable linear and logistic regression models.

**Results:**

Primary 877 ARDS patients (mean ± standard deviation: 54 ± 16 years, 68% male) survived and were discharged from ICU. Out of these patients, 478 (54.5%) were tracheotomised during ICU treatment. After 12 months, patient-reported outcomes could be analysed of 388 (44.2%) respondents, 205 with tracheostomy and 183 without. One year after ICU discharge, tracheostomy showed no significant association with physical or mental health-related quality of life (PCS-12: − 0.73 [− 3.96, 2.51]; MCS-12: − 0.71 [− 4.92, 3.49]), symptoms of psychiatric disorders (depression: 0.10 [− 1.43, 1.64]; PTSD: 3.31 [− 1.81, 8.43]; anxiety: 1.26 [0.41, 3.86]; obsessive–compulsive disorder: 0.59 [0.05, 6.68]) or return-to-work (0.71 [0.31, 1.64]) in the multivariable analysis (OR [95%-CI]).

**Conclusions:**

Up to 1 year after ICU discharge, neither HRQoL nor symptoms of psychiatric disorders nor return-to-work was affected by tracheostomy.

*Trial registration* NCT02637011 (ClinicalTrials.gov, Registered 15 December 2015, retrospectively registered)

## Introduction

Acute respiratory distress syndrome (ARDS), a life-threatening condition characterised by direct or indirect damage to the lung parenchyma causing critical hypoxemia with or without hypercapnia [1]. Healthcare research in critical care medicine is a relatively new field of interest focusing on the influence of organisational structures, the processes of care on mortality and health-related quality of life (HRQoL) of intensive care unit (ICU) survivors [2–4]. ARDS survivors experience substantial long-term limitations on their physical and mental health and have a high risk of developing psychiatric disorders [5–7].

As part of the complex treatment of ARDS, patients often need prolonged mechanical ventilation. Prolonged endotracheal intubation results in acute laryngeal injuries in more than half of patients and is associated with significantly worse breathing and voicing up to 10 weeks after extubation [8]. Tracheostomy (TT), as the most commonly performed procedure in mechanically ventilated ICU patients, may not only increase patient comfort by reducing airway resistance and dead space but also avoids potential intubation injuries such as oropharyngeal and laryngeal lesions. The advantages of TT over conventional intubation are a shorter duration of sedation and thus promotion of early mobilisation and reduced work of breathing, which might facilitate weaning from mechanical ventilation and hence oral food intake [9]. Nevertheless, the TT is an invasive treatment with potential life-threatening risks. Various complications (e.g. haemorrhage, infection, pneumothorax, tube obstruction, accidental decannulation) have been reported in up to 15% of ICU patients [10, 11]. A systematic review described that the most common causes of TT-related deaths were haemorrhage, loss of airway and false passage [12]. In the United States, TT results in death or permanent disability in nearly 500 patients each year [11].

To date, outcomes’ research in TT has focused on clinical outcomes such as ventilator-free days, duration of sedation, length of stay in the ICU, mortality or survival [13–15]. Patient-reported outcomes such as HRQoL or patient-reported symptoms of psychiatric disease have not been investigated so far, nor has return-to-work. Also, a long-term follow-up perspective is missing.

Therefore, the aim of this study was to analyse the association of TT with HRQoL, the prevalence of psychiatric symptoms and return-to-work of ARDS survivors up to 1 year after ICU discharge. Our hypotheses were that there are possible negative associations between TT and HRQoL, the prevalence of psychiatric symptoms and return-to-work of ARDS survivors.

## Methods

### Study design

The frequency of TT in patients with ARDS was observed in the context of a large prospective German-wide cohort study (DACAPO, acronym of study title: Surviving ARDS: the influence of quality of care and individual patient characteristics on quality of life, ClinicalTrials.gov Identifier: NCT02637011). The study was approved by the Ethics Committee of the University of Regensburg (original approval: December 2013, approval of an amendment: June 2014; file number 13-101-0262) and (if necessary) by the Ethics Committees of the participating study centres. The baseline characteristics and profile of the cohort have been described in more detail elsewhere [16, 17]. ARDS survivors were asked to complete comprehensive self-report questionnaires 3, 6 and 12 months after their discharge from the ICU.

### Patient cohort

**Fig. 1:**
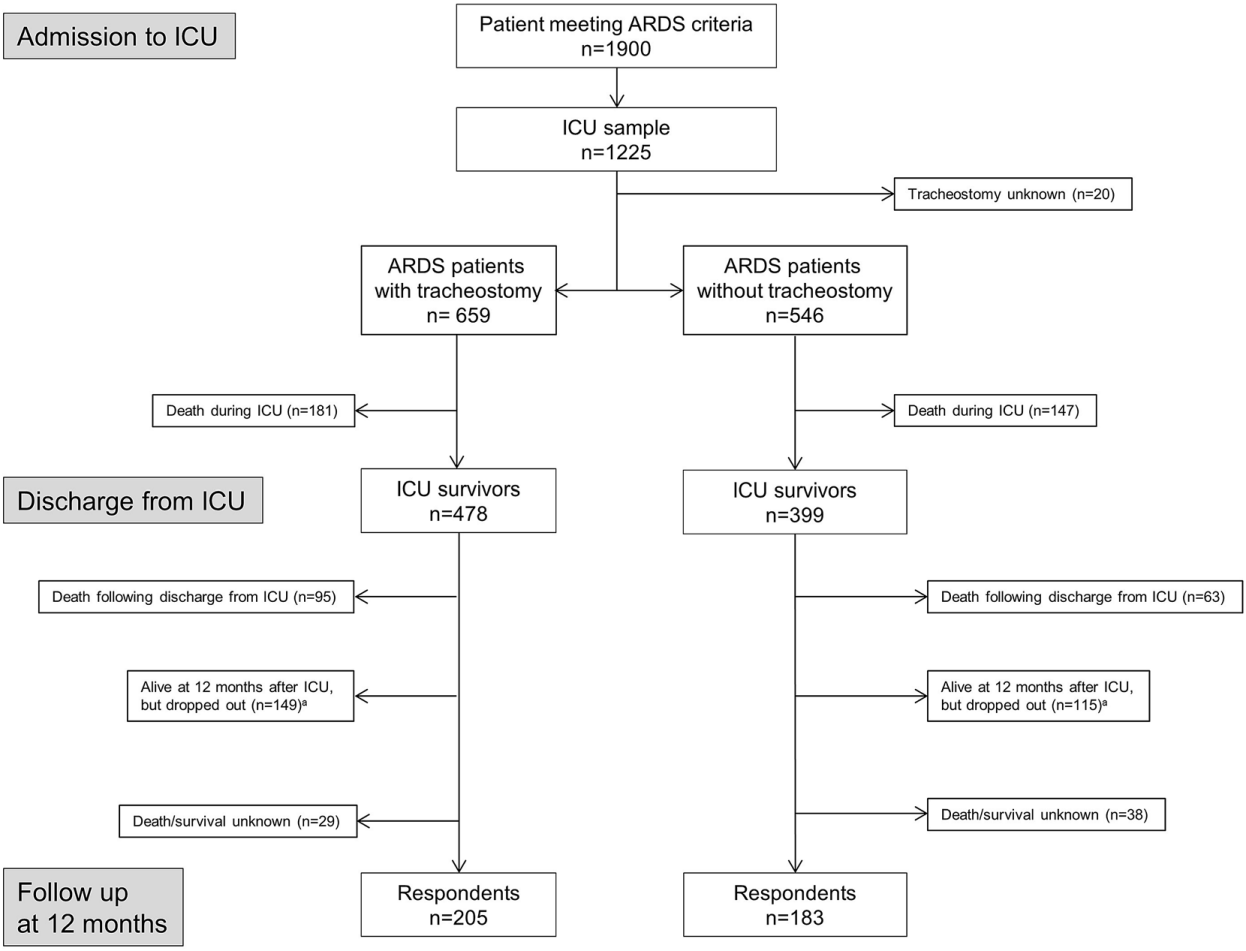
Consort statement—flow chart of ARDS patients throughout the study (^a^reasons for drop-out for all patients: unable to participate in the study or to understand German, declined participation; incorrect address or new address unknown, organisational failure; survival was assessed by means of local municipal population registries)

Figure 1 gives an overview of the sample size at the different time points of the study. The study preliminarily included patients with ARDS (according to the Berlin definition [18]) who had been treated at one of 61 ICUs across Germany between September 2014 and April 2016. Written informed consent was obtained from 1.225 patients. Written informed consent was given by the patients or their caregivers or legal guardians during the ICU length of stay.

Out of 877 ICU survivors, 388 (44.2%) had returned the questionnaire at 12 months. The most frequent reason for dropping out of the study was death after discharge from the ICU (*N* = 158). Other reasons included inability to complete the questionnaire (insufficient knowledge of German or incapability due to morbidity), absence of a person who could provide proxy reports, withdrawal of consent or an invalid address.

The primary endpoint of this study was HRQoL (Short Form-12 self-report questionnaire [SF-12]) of ARDS survivors up to 1 year after ICU discharge. Secondary endpoints were the prevalence of psychiatric symptoms and return-to-work up to 1 year after ICU discharge.

### Data collection and measurement instruments

Characteristics of patients (age, sex), disease (Sequential Organ Failure Assessment [SOFA] Score, Simplified Acute Physiology Score [SAPS II] and ARDS severity) and treatment (TT, prone position and extracorporeal membrane oxygenation) as well as information on mortality or discharge from the ICU were reported by study physicians or study assistants of the participating ICUs using the electronic data capture system OpenClinica (OpenClinica, LLC; https://www.openclinica.com/). Information on HRQoL and return-to-work was assessed by means of self-report questionnaires at 3, 6 and 12 months after discharge from the ICU. ARDS survivors completed the Short Form-12 self-report questionnaire (SF-12) as a measure of HRQoL and questionnaires on psychopathology. The prevalence of psychiatric symptoms (depression and post-traumatic stress disorder [PTSD]) was assessed using the Patient Health Questionnaire-9 [PHQ-9] and the Post-Traumatic Stress Syndrome-14 [PTSS-14]. Furthermore, patients were asked whether depression, PTSD, anxiety or obsessive compulsive disorder (OCD) had been diagnosed by a physician after ICU discharge. For the screening tools, the cut-off values for being at risk of depression were defined as PHQ-9 (≥ 5) and for PTSD as PTSS-14 [≥ 45]) [19, 20]. The published scoring algorithm of the SF-12 resulted in the Physical Component Summary (PCS-12) and the Mental Component Summary (MCS-12) scores [21]. Scores range from 0 to 100 (higher values indicate better HRQoL), and a score of 50 represents the mean value for the general population (German norm values) [22, 23]. The measuring instruments of HRQoL are described in detail elsewhere [16].

### Statistical analyses

The analyses presented here were pre-specified at the design stage of the study. Data are shown as mean ± SD for continuous and as absolute and relative frequencies for categorical variables. The Student’s *t* test and the Chi squared test of independence were used for comparing tracheotomised and non-tracheotomised patients. We tested whether there was systematic variance between ICUs in relation to the primary outcome at the three follow-ups. These analyses yielded an intraclass correlation coefficient (ICC) close to zero based on the fully unconditional model and non-significant *p* values for the likelihood ratio tests. For this reason, fixed-effects linear and logistic regression models were applied. The predictive value of TT on HRQOL, psychiatric symptoms and return-to-work after 12 months were assessed by means of multiple linear and multivariable logistic regression models. The following patient- and treatment-related confounders were used: patient age, sex, SOFA Score, SAPS II, ARDS severity, body mass index (BMI) and length of ICU stay. The regression coefficient B for the linear regression models and the OR for the logistic regression model were calculated as effect estimates, both accompanied by the corresponding 95%-confidence intervals (95%-CI). A *p* value of < 0.05 was considered significant. Due to the exploratory nature of this study, no adjustments for multiple comparisons were made. All analyses were performed using the software R (Version 3.5.1, http://www.r-project.org).

## Results

Out of 1900 patients meeting ARDS criteria, 1205 patients could be analysed (Fig. 1). Of whom 659 (54.7%) had been tracheotomised during ICU treatment. 68% of this patient cohort were men; the mean age was 56 years (± 15.8). The characteristics of patients with and without TT are shown in Table 1. The body mass index (BMI) was significantly higher in the group of tracheotomised patients (*p* = 0.010). The two groups did not differ with regard to illness severity, which was measured by the SAPS II at ICU admission, the SOFA score and ARDS severity at the time of ARDS diagnosis. For all 1205 patients, the median duration of mechanical ventilation was 16 days (IQR 10–27) and that of ICU length of stay 22 days (IQR 14–36). The median duration of ventilation (20 days [IQR 13–29] vs. 11 days [IQR 6–16], *p* < 0.001) and ICU stay (*p* < 0.001, Table 1) was twice as long for tracheotomised patients. Primary 877 ARDS patients survived and discharged from ICU. Out of these patients 478 (54.5%) were tracheotomised.

**Table 1:** Patient characteristics of the ICU sample (*n* = 1205)

	Tracheostomy (*n* = 659)	Non-tracheostomy (*n* = 546)	*p* value	Missing (*n*)
Age (years), mean (± SD)	56.41 (± 14.87)	56.01 (± 16.89)	0.663	0
Sex				
Men, n (%)	454 (69%)	368 (67%)	0.580	0
Women, *n* (%)	205 (31%)	178 (33%)		
BMI (kg/m^2^), mean (± SD)	29.62 (± 9.34)	28.29 (± 6.94)	0.010*	154
Severity of ARDS^a^				
Mild, *n* (%)	46 (9%)	44 (10%)	0.711	273
Moderate, *n* (%)	224 (44%)	182 (42%)		
Severe, *n* (%)	238 (47%)	211 (48%)		
SAPS^b^, mean (± SD)	65 (± 41.36)	51 (± 41.02)	0.655	124
SOFA score^a^, mean (± SD)	8.46 (± 3.58)	8.10 (± 3.34)	0.098	148
Duration of ICU stay, days, median (IQR)	30 (20–44)	15 (10–23)	< 0.001*	44

*ARDS* acute respiratory distress syndrome, *BMI* body mass index, *ICU* intensive care unit, *SAPS* Simplified Acute Physiology Score, *SOFA* sequential organ failure assessment

*p < 0.05

^a^Measured at the time of ARDS diagnosis

^b^Measured at ICU admission

The employment status of ARDS patients before and after ICU treatment is shown in Table 2. 328 (43.3%) ARDS survivors had worked full-time or part-time before their critical illness. After 1 year, 186 (54.2%) of the respondents had returned to work within a median time of 114 days (IQR 72–190) after ICU discharge. Univariate analysis showed that tracheotomised patients were less likely to have returned to work after 12 months than non-tracheotomised patients (35% vs. 64%, *p* < 0.001). This association could not be shown in the multivariable analysis (*p* = 0.416) (see Table 4).

**Table 2:** Univariate analysis of the employment status of ARDS patients before ICU treatment and return-to-work

	Tracheostomy	Non-tracheostomy	*p* value	Missing (n)
ICU sample (*n* = 1205)				
Previous employment, *n* (%)	211 (36.1)	197 (40.6)	0.133	136
No previous employment, *n* (%)	373 (63.9)	288 (59.4)		
ICU survivors (*n* = 877)				
Previous employment, *n* (%)	162 (39)	166 (48.4)	0.01*	119
No previous employment, *n* (%)	253 (61)	177 (51.6)		
Respondents after 12 months (*n* = 388)				
Return-to-work, *n* (%)	34 (35)	66 (64)	*p* < 0.001*	189

Return-to-work could only analysed in patients which worked before the critical illness

*ICU* intensive care unit

*p < 0.05

**Fig. 2:**
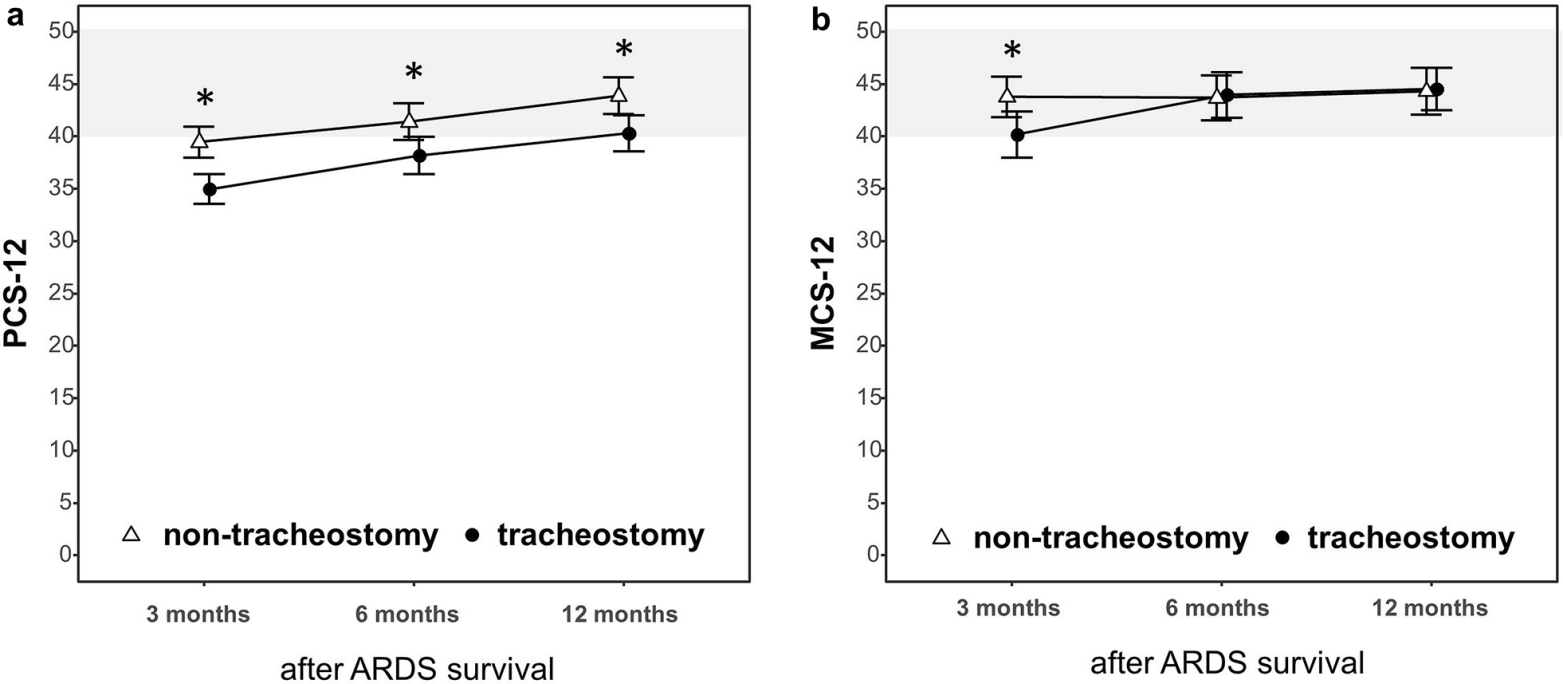
Univariate analysis of Short Form-12 (SF-12) self-report response of ARDS survivors across follow-up time points (*p < 0.05; means and 95%-CI). **a** Physical Component Summary of SF-12 [PCS-12]. **b** Mental Component Summary of SF-12 [MCS-12]); the mean score of the general population is 50; one standard deviation is marked in grey

According to SF-12 measurement, ARDS survivors had lower HRQoL compared to the score validated from the general population. The differences in PCS-12 and MCS-12 between ARDS survivors with and without TT across the follow-up time points are shown in Fig. 2. In the univariate analysis, patients with TT had significantly worse PCS-12 scores than non-tracheotomised patients across all measured time points after ICU discharge (Fig. 2; Table 3), but no significance was found in the multivariable analysis (Table 4). In the univariate analysis, MCS-12 scores were significantly impaired for tracheotomised patients 3 months after ICU discharge (*p* = 0.016) but did not differ between the two groups after 6 and 12 months (Fig. 2). The multivariable analysis of MCS-12 did not show any differences 1 year after ICU discharge (*p* = 0.834) (Table 4). In the univariate and multivariate analyses, psychiatric symptoms (PHQ-9, PTSS-14) and reported psychiatric disorders (depression, PTSD, anxiety and OCD) had not been affected by TT in patients up to 1 year after ARDS (Tables 3 and 4).

**Table 3:** Univariate analysis of HRQoL and symptoms of psychiatric disorders in ARDS survivors after 12 months (*n* = 388)

	Tracheostomy (*n* = 205)	Non-tracheostomy (*n* = 183)	*p* value	Missing (n)
PCS-12^a^, mean (± SD)	40.3 (± 10.8)	43.9 (± 11.0)	0.005*	83
MCS-12^a^, mean (± SD)	44.5 (± 12.8)	44.3 (± 14.0)	0.887	83
PHQ-9^a^, mean (± SD)	6.5 (± 5.1)	6.1 (± 5.4)	0.517	24
PTSS-14^a^, mean (± SD)	35.3 (± 18.0)	32.6 (± 17.6)	0.156	25
Depression^b^				
Yes, *n* (%)	32 (16%)	18 (10%)	0.078	18
Anxiety disorder^b^				
Yes, *n* (%)	20 (10%)	11(6%)	0.170	34
PTSD^b^				
Yes, *n* (%)	20 (10%)	16 (9%)	0.721	22
OCD^b^				
Yes, *n* (%)	6 (3%)	5 (3%)	0.883	27

*MCS-12* Mental Component Scale of Short-Form 12 Questionnaire, *PCS-12* Physical Component Scale of Short-Form 12 Questionnaire, *OCD* obsessive–compulsive disorder, *PTSD* post-traumatic stress disorder, *PHQ-9* Patient Health Questionnaire-9, *PTSS-14* Post-Traumatic Stress Syndrome 14-Questions Inventory

*p < 0.05

^a^Health-related quality of life and symptoms of psychiatric disorders (depression, PTSD) were diagnosed according to the results of patient self-reported questionnaires

^b^Psychiatric disorders diagnosed by a physician were recorded by patient self-report in the follow-up questionnaires

**Table 4:** Multivariable linear and logistic regression models on HRQoL, psychiatric symptoms and return-to-work after 12 months (adjusted for age, sex, BMI, severity of ARDS, SOFA score, SAPS-II and length of ICU stay)

Dependent variable	Independent variable	B (95%-CI)	p
PCS-12^a^ (*n* = 305)	Tracheostomy	− 0.73 (− 3.96, 2.51)	0.659
MCS-12^a^ (*n* = 305)	Tracheostomy	− 0.71 (− 4.92, 3.49)	0.739
PHQ-9^a^ (*n* = 364)	Tracheostomy	0.10 (− 1.43, 1.64)	0.894
PTSS-14^a^ (*n* = 363)	Tracheostomy	3.31 (− 1.81, 8.43)	0.204

		OR (95%-CI)	
Depression^b^ (*n* = 370)	Tracheostomy	1.52 (0.61, 3.78)	0.368
Anxiety disorder^b^ (*n* = 354)	Tracheostomy	1.26 (0.41, 3.86)	0.691
PTSD^b^ (*n* = 366)	Tracheostomy	0.91 (0.29, 2.82)	0.870
OCD^b^ (*n* = 361)	Tracheostomy	0.59 (0.05, 6.68)	0.667
Return-to-work^b^ (*n* = 199)	Tracheostomy	0.71 (0.31, 1.64)	0.416

*B* regression coefficient, *95%-CI* 95% confidence interval, *MCS-12* Mental Component Scale of Short-Form 12 Questionnaire, *OCD* obsessive–compulsive disorder, *OR* odds ratio, *PCS-12* Physical Component Scale of Short-Form 12 Questionnaire, *PTSD* post-traumatic stress disorder, *PHQ-9* Patient Health Questionnaire-9, *PTSS-14* Post-Traumatic Stress Syndrome 14-Questions Inventory

*p < 0.05

^a^Health-related quality of life and symptoms of psychiatric disorders (depression, PTSD) were diagnosed according to the results of patient self-reported questionnaires

^b^Psychiatric disorders diagnosed by a physician were recorded by patient self-report in the follow-up questionnaires

## Discussion

This study analysed the influence of TT on HRQoL, prevalence of psychiatric symptoms and return-to-work in 388 ARDS survivors 1 year after ICU discharge. The main finding was that TT was not significantly associated with physical (PCS-12) or mental impairment in quality of life (MCS-12). Secondary findings were (1) TT was not significantly related to the prevalence of psychiatric disorders and (2) TT was not significantly associated with return-to-work of ARDS survivors after 12 months.

Thus, the hypotheses underlying this study could not be confirmed. However, we did observe that TT was associated with a significant increase of ICU length of stay. ICU length of stay is a known independent factor associated with increased stationary healthcare use and 1-year mortality after ICU discharge and should be considered when deciding for the best time point of TT in critical ill patients [24, 25]. In our study, associations observed in univariate analyses were lost mainly due to the strong confounding effect of ICU length of stay.

A current systematic review of patient-important outcomes for critically ill patients in randomised controlled clinical studies has shown ICU mortality to be the most frequently measured clinical outcome. Only 10% of studies have included at least one patient-important outcome in addition to mortality after ICU discharge [26]. It should be noted that, in contrast to mortality, HRQoL is a complex construct that contains individual aspects with multiple dimensions, often operationalised as social, somatic and psychological variables [4]. The individual dimensions of HRQoL could be recorded by different measurement tools and must be correlated for a better comparison of patient-reported outcomes [27]. In an Italian study, 137 tracheotomised patients with respiratory and neurological diseases were analysed in relation to ICU mortality and HRQoL. Patients tracheotomised because of respiratory disease had a high ICU mortality rate of 50%; only 25 patients (20.5%) were still alive after 1 year. HRQoL of all tracheotomised patients was moderately compromised after 1 year, but the group of patients with respiratory diseases showed better HRQoL during follow-up [28]. In the present study, HRQoL was not impaired by TT.

Symptoms of psychiatric disorders were often reported after survival of ARDS [6, 29]. As an example, up to 27% of critical illness survivors suffered from PTSD, and depression was reported in 40% of ARDS survivors [30, 31]. In the present study, TT did not influence the occurrence of psychiatric disorders. According to the results of the self-reported questionnaires shown in Tables 3 and 4, 25.9% of ARDS survivors had symptoms of PTSD (cut-off scores for symptoms of PTSD: ≥ 45) and 55.6% symptoms of depression (PHQ-9 score ≥ 5) at the 1-year follow-up [19, 20].

Furthermore, TT did not affect return-to-work in ARDS survivors in this study. More than 50% of 1-year survivors had returned to work after a median of 16 weeks after ICU discharge. Considering the different socioeconomic systems, an US survey including 379 previously employed ARDS survivors found that 213 (56%) patients had returned to work after a median of 13 weeks after hospital discharge [32]. Nevertheless, the fact remains that nearly 50% of patients were unemployed, which may have resulted in family problems and loss of substantial earnings. A 5-year follow-up of ARDS survivors showed that nearly one-third of previously employed ARDS survivors had never returned to work [33].

TT is one of the most common surgical procedures in patients with acute respiratory failure. TT practices vary substantially amongst disciplines, ICUs and institutions [34]. The TT rate of 55% found in this Germany-wide study seems to be rather high and could be explained by the inclusion of a higher number of patients with severe ARDS. In the worldwide LUNG-SAFE study, only 13% of patients with ARDS had received TT during their length of ICU stay; yet, the rate in European countries was higher at 15.7% [15]. The ICU length of stay was also significantly longer for patients with TT (11 vs. 8 days). One possible explanation might be that the transfer of tracheostomised patients to a normal ward is difficult because of the elaborate airway management and more intensive care. Freeman and colleagues analysed over 44,000 patients with acute respiratory failure (10.8% of patients with TT) and found longer ICU treatment (24 vs. 7 days) in tracheotomised patients [35]. In our study, the BMI was significantly higher in the tracheotomised group. A recent study from the US which also investigated patient factors associated with 30-day survival after TT did not find any influence of BMI and socioeconomic factors [13].

### Strengths and limitations

The strengths of the present study are its prospective design with three time point of follow-ups, the large number of included patients with ARDS from hospitals across Germany and the detailed collection of data on HRQoL and individual patient characteristics. Despite our best efforts to follow up each patient, the number of drop-outs was rather high, which may have resulted in attrition bias limiting this study. The instruments used for screening mental disorders do not allow making diagnoses such as major depression disorders and PTSD; only symptoms or the risk of being affected by such a disorder could be recorded. To the best of our knowledge, this is the largest multicentre study investigating the association of TT with return-to-work and patient-reported HRQoL as well as symptoms of psychiatric disorders and return-to-work in ARDS survivors.

## Conclusion

In summary, TT resulted in prolonged intensive care treatment of ARDS survivors but did not seem to have an association with physical or mental HRQoL, symptoms of psychiatric disorders or return-to-work 1 year after ICU discharge.

## Data Availability

Data are available depending permission from the original data holders (the two principal investigators (PIs) Christian Apfelbacher and Thomas Bein). Due to data economy and subsequently data protection, only the variables required for the analysis project will be provided.

## Abbreviations

ARDS: Acute respiratory distress syndrome
BMI: Body mass index
HRQoL: Health-related quality of life
ICU: Intensive care unit
MCS-12: Mental Component Summary
OCD: Obsessive compulsive disorders
PCS-12: Physical Component Summary
PHQ-9: Patient Health Questionnaire-9
PTSD: Post-traumatic stress disorders
PTSS-14: Post-Traumatic Stress Syndrome 14-Questions Inventory
SAPS II: Simplified Acute Physiology Score
SF-12: Short Form-12 self-report questionnaire
SOFA: Sequential organ failure assessment
TT: Tracheostomy
